# Directed Immobilization of Horseradish Peroxidase Using a Covalently Attached Competitive Inhibitor

**DOI:** 10.3390/molecules31152669

**Published:** 2026-07-31

**Authors:** Anne Muschter, Sophia Rosencrantz, Takwa Chouki, Stefan Reinicke, Ruben R. Rosencrantz

**Affiliations:** 1 Division of Life Science & Bioprocesses, Fraunhofer Institute for Applied Polymer Research IAP, Geiselbergstr. 69, 14476 Potsdam, Germany; 2 Chair for Biofunctional Polymer Materials, Brandenburg University of Technology (BTU) Cottbus-Senftenberg, Universitätsplatz 1, 01968 Senftenberg, Germany; 3 Fraunhofer Cluster of Excellence Immune-Mediated Diseases CIMD, 60528 Frankfurt am Main, Germany

**Keywords:** site-directed immobilization, horseradish peroxidase, competitive inhibitor, enzyme immobilization, biocatalysis

## Abstract

We developed a site-directed immobilization strategy exploiting the competitive inhibitor Remazol Brilliant Blue R (RB) to orient horseradish peroxidase (HRP) on functionalized glass surfaces. RB was covalently bound to poly(ethylene-alt-maleic anhydride) (PEMA)-coated substrates, enabling HRP binding via its active site. Subsequent contact with a second PEMA-functionalized surface facilitated covalent immobilization of HRP with controlled orientation. Interfacial layer assembly and the comparative random control were characterized by AFM and XPS analyses, while successful RB acetylation was confirmed by NMR and ATR-IR spectroscopy. Directed contact approaches using stepwise RB and PEMA+RB functionalization yielded HRP loadings with specific activities approximately twofold higher than non-directed immobilization. Our inhibitor-mediated method preserves the native enzyme structure without genetic modification, offering a straightforward platform for enhancing enzyme performance. This strategy is promising for applications in biosensors, biocatalysis, and enzyme-based devices requiring efficient enzyme immobilization.

## 1. Introduction

Enzymes are efficient and selective catalysts that play key roles in biological systems and industrial processes. For instance, peroxidases are commonly present in biological systems to eliminate reactive oxygen species, which can be formed during aerobic cellular respiration and other processes, thereby preventing damage to healthy cells [1,2,3,4,5]. Immobilization is widely used to improve enzyme economic efficiency compared to solution-based use [6,7,8], as it enhances structural rigidity and stability under harsh conditions [7,9,10]. This approach also enables enzyme reuse across multiple reaction cycles and facilitates integration into continuous processes [8,11,12]. Several approaches have been developed for enzyme immobilization [13], including physical adsorption [14], covalent attachment [15], encapsulation [16], and cross-linking [17]. Among these, covalent coupling is particularly attractive as it provides a stable and irreversible linkage [18]. However, conventional covalent immobilization methods typically rely on the naturally occurring functional groups of amino acid side chains (such as –NH_2_, –COOH, or –SH) and are applied in a largely random manner. This lack of control can obstruct enzyme active sites or alter the enzyme’s three-dimensional structure, often resulting in reduced catalytic activity [19,20,21,22,23,24,25]. Therefore, site-specific immobilization of enzymes has emerged as an effective strategy to control active-site orientation and enhance catalytic performance. A variety of site-specific enzyme immobilization techniques have been developed, including approaches based on genetic fusion tags, bio-orthogonal ligation systems, and non-standard amino acid (NSAA) incorporation [26]. These methods enable precise control over protein binding orientation, helping to preserve their active conformations and catalytic function [27,28].

For instance, SpyTag/SpyCatcher (Spy-chemistry) allows site-specific immobilization and protein purification, in which proteins are equipped with SpyTags and resins with SpyCatchers [29,30]. Similarly, SNAP-tag technology has been used for directed immobilization onto benzylguanine-activated sepharose, yielding affinity columns with up to three-fold higher binding capacities than non-directed coupling [31]. In another approach, bacteriophage-derived magnetic nanoparticles were developed for site-specific immobilization of cytidine deaminase, yielding a biocatalyst with high catalytic efficiency and full activity retention over 10 cycles [32]. Similarly, site-specific immobilization to attach bilirubin oxidase was employed via a single engineered cysteine to maleimide-functionalized electrodes, enabling controlled orientation, stable attachment and retention of catalytic activity [33]. Site-specific immobilization has also been demonstrated for glucose-sensing proteins on silica nanoparticles using protein fusion-tags or DNA-directed methods, which outperformed non-directional cross-linking and enabled efficient nanosensor generation for metabolite detection [34]. Beyond these examples, numerous other site-specific strategies have been explored, employing diverse biochemical coupling systems and surface functionalization approaches to optimize enzyme orientation, stability, and catalytic efficiency [35,36,37,38,39,40].

**Figure 1:**
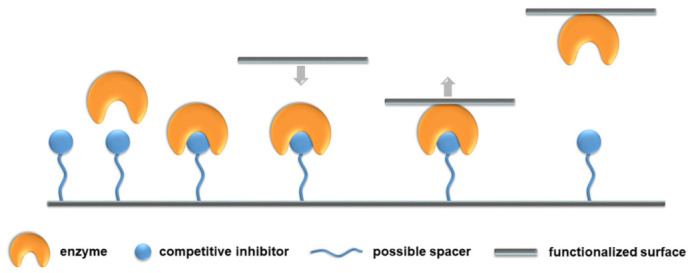
Schematic illustration of site-directed, inhibitor-mediated enzyme immobilization.

While these methods have demonstrated significant improvements, many still rely on genetic modification [26,29,31,32], specialized reagents [30,33], or complex preparation steps [34,35]. To address these challenges, we developed an inhibitor-mediated, directed immobilization strategy, which was schematically illustrated in Figure 1. In this approach, competitive inhibitors specific to the target enzymes are first covalently bound to an appropriate surface. The enzyme then binds to the immobilized inhibitor via its active site. Subsequently, it is transferred to a second functionalized surface through a contact process, enabling covalent attachment in this oriented configuration while leaving the active site freely accessible. This strategy improves the activity of covalently bound enzymes on surfaces [41]. Additionally, this method does not require genetic modification, allowing the use of native proteins.

In this study, the model enzyme horseradish peroxidase (HRP) from *Armoracia rusticana* was used. HRP is highly relevant across laboratory and industrial sectors due to its extensive use as a sensitive reporter label in high-sensitivity immunodiagnostics, enzyme-linked immunosorbent assays (ELISA), Western blotting, and advanced biosensor development [42]. This widespread application is reflected in its expanding global market footprint. Market evaluations by Global Market Insights and Mordor Intelligence value the global HRP sector at approximately USD 65–72 million, with projections expected to exceed USD 110 million by 2030–2032 at a compound annual growth rate (CAGR) of 7.0–8.5% [43,44,45,46]. This projected expansion is driven by increasing demands for automated assay workflows, point-of-care testing devices, and emerging biotechnological applications [47]. Together, these trends underscore the significant technological and economic relevance of HRP.

**Figure 2:**
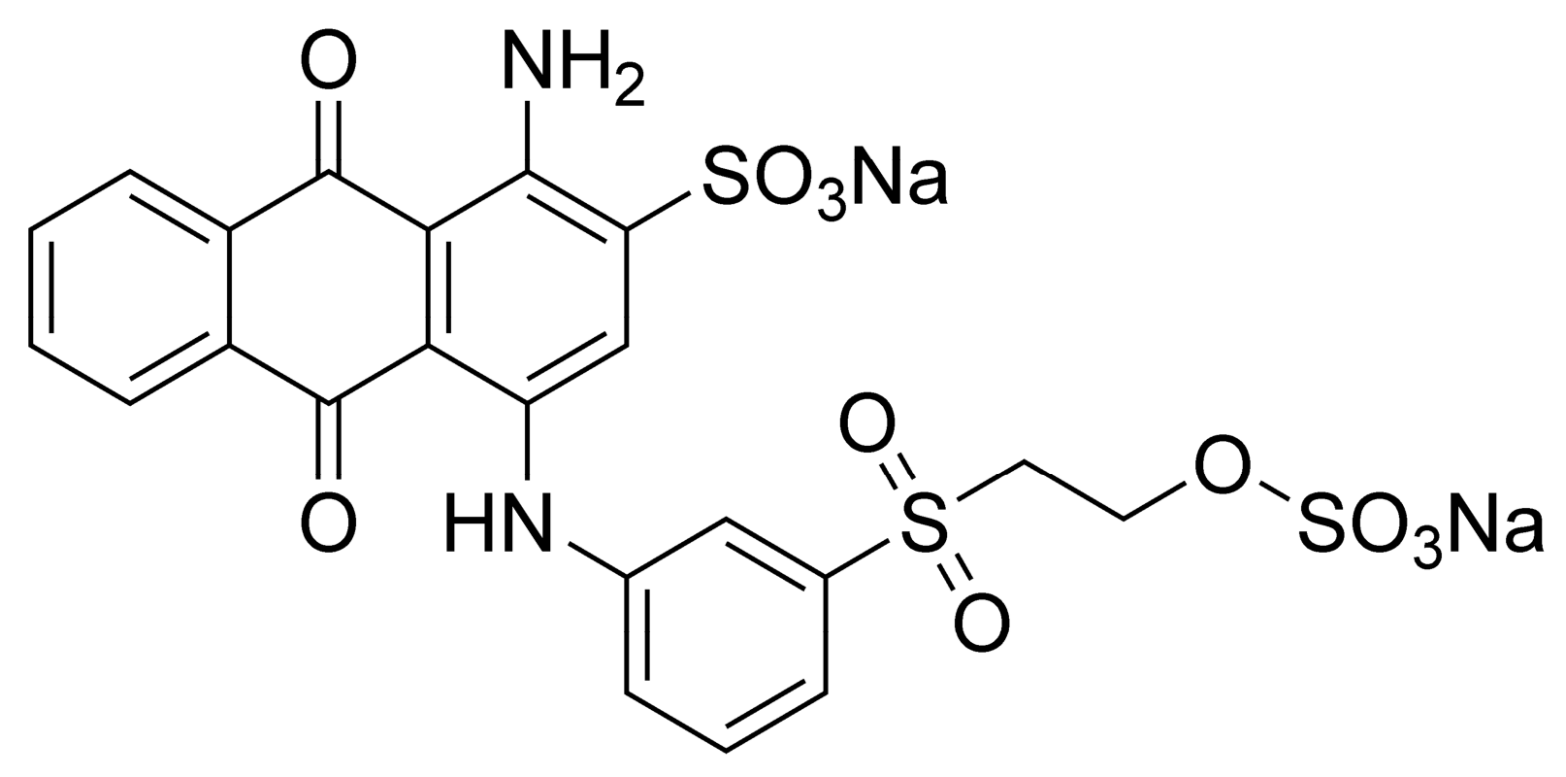
Chemical structure of Remazol Blue.

The anthraquinone dye Remazol Brilliant Blue R (RB, Figure 2) has proven to be an effective competitive inhibitor of HRP [48]. RB inhibits HRP at pH values above six and is partially degraded as a substrate at lower pH values in the presence of an equimolar amount of hydrogen peroxide [48]. The inhibitory effect of the dye has also been described when using soybean peroxidase, although it is relatively weak [49].

In this work, glass substrates, specifically cover slips and glass beads, were used as carrier materials. These were pretreated oxidatively, followed by amino-silanization and subsequent binding of a functional copolymer [50,51]. Poly(ethylene-alt-maleic anhydride) (PEMA) was chosen as the copolymer due to its reactive maleic anhydride units, which readily react with nucleophiles such as amines [52]. Additionally, PEMA swells in the presence of water, creating thicker, more voluminous surface layers [53,54,55]. Building upon this platform, we developed an inhibitor-mediated strategy for site-directed covalent immobilization of HRP that differs fundamentally from existing approaches. Instead of relying on protein engineering or external affinity tags, our method employs a competitive inhibitor to transiently bind the enzyme at its active site, thereby defining its orientation. This predefined orientation is subsequently preserved through a two-surface contact transfer process, enabling covalent attachment while maintaining active-site accessibility. To the best of our knowledge, this combination of inhibitor-mediated orientation using RB and contact-based immobilization represents a novel strategy that has not been reported previously. The approach requires only the native enzyme and commercially available reagents, providing a simple and effective alternative to established site-specific immobilization strategies.

## 2. Results and Discussion

### 2.1. Surface Characterization of the Matrix

**Figure 3:**
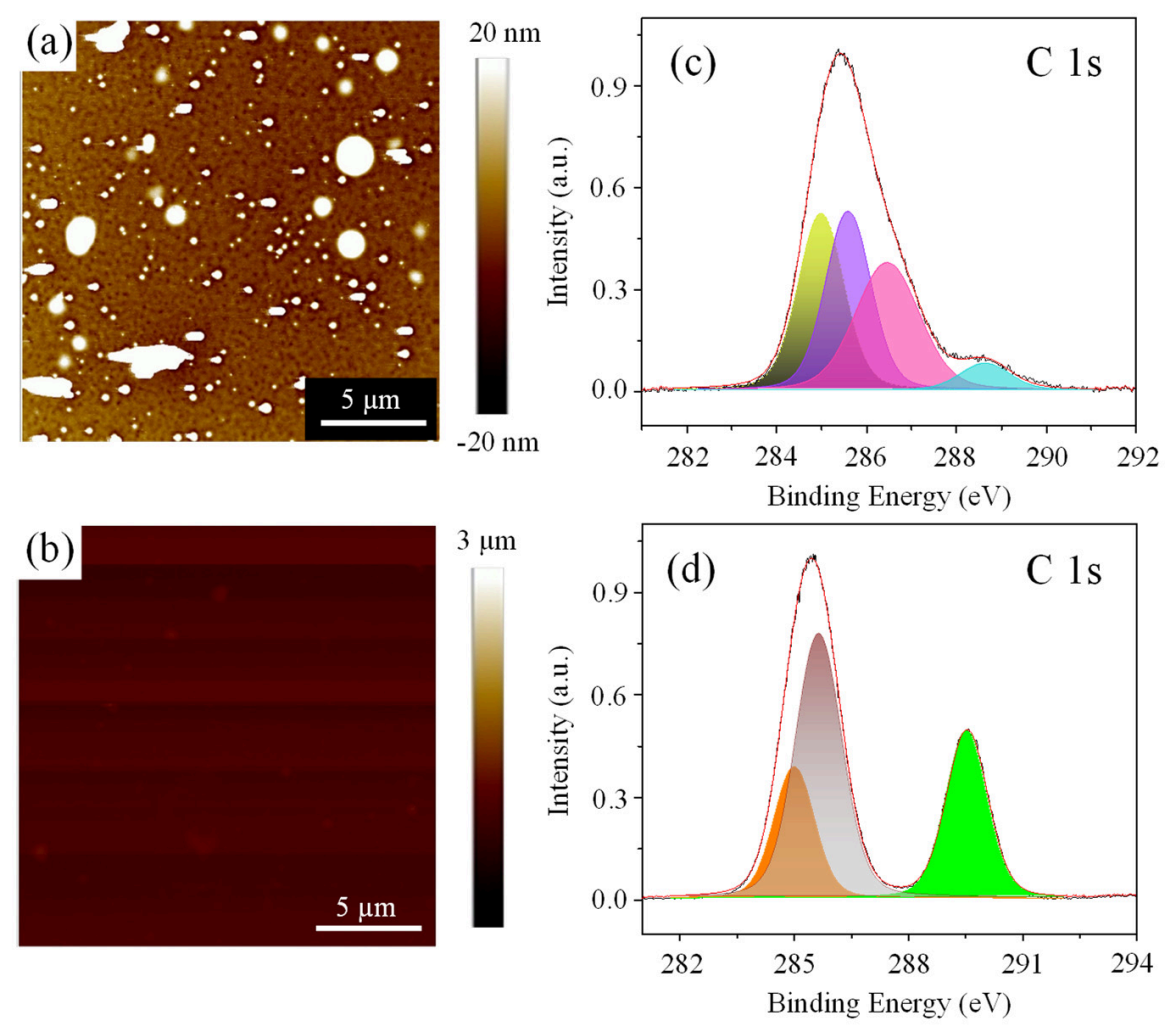
AFM and XPS images of the sequentially functionalized glass substrates. AFM images show the surface morphology of (**a**) the APTES layer, (**b**) the PEMA coating. High-resolution XPS C 1s spectra are shown for (**c**) APTES, and (**d**) PEMA illustrating progressive chemical modification of the substrate.

To monitor the surface modification steps, the substrates were characterized by atomic force microscopy (AFM) and X-ray photoelectron spectroscopy (XPS) (Figure 3). AFM was employed to assess the evolution of surface morphology during the sequential functionalization of the glass substrates [56]. Following aminosilanization with (3-Aminopropyl)triethoxysilane (APTES) (Figure 3a), the surface exhibited a heterogeneous topography characterized by protruding spherical features and larger irregular aggregates distributed across the substrate. Such features are frequently observed for silane-treated surfaces and may arise from silane self-condensation and oligomerization [57,58]. After deposition of the PEMA layer (Figure 3b), the surface became more homogeneous, with the prominent features observed on the APTES-coated substrate largely obscured by the polymer film, indicating effective coverage of the underlying silanized substrate. These morphological changes are further supported by surface chemical analysis using XPS, which provides complementary evidence for the successive assembly of the interfacial layers [59]. The survey spectra confirmed the expected layer-by-layer evolution of the supports (Table S1). The APTES-modified substrate showed a pronounced Si 2p signal (15.73 at.%) and a clear N 1s contribution (6.84 at.%), consistent with silane grafting and the presence of terminal amino groups [60,61]. The corresponding C 1s spectrum (Figure 3c) is characterized by a broad envelope centered near 285.5 eV. Deconvolution of this envelope resolves the signal into its underlying sub-peak components: an aliphatic (C–C/C–H) contribution resolved at 285.0 eV and a shifted aliphatic/spacer contribution at 285.6 eV [62]. Additional higher-binding-energy contributions attributable to C–N and C–O species are observed at 286.6 eV [62]. After deposition of the PEMA layer, the surface became strongly carbon- and oxygen-rich (with C 1s increasing to 66.33 at.%), and O 1s increasing to 33.11 at.%), while the Si 2p and N 1s signals were markedly attenuated down to 0.52 at.% and 0.03 at.%, respectively, consistent with effective coverage of the underlying substrate. The C 1s spectrum of the polymer layer (Figure 3d) showed an envelope consistent with the expected chemical composition of PEMA [63]. This included an aliphatic (C–C/C–H) contribution centered around 285.0 eV, a component attributed to carbon atoms adjacent to anhydride groups around 285.9 eV, and a higher-binding-energy anhydride carbonyl component near 289.6 eV [63]. Together with the AFM findings, these results provide complementary morphological and chemical evidence for successful layer-by-layer assembly.

### 2.2. Characterization of RB and Its Derivative

Acetylation of RB was carried out to investigate the influence of the primary amino group on the inhibitory effect of RB on HRP activity. Although acetylation with acetyl chloride served here as a model reaction to confirm the nucleophilicity of RB’s amino groups, it acts as a proxy rather than a full mimic of the reaction with PEMA’s maleic anhydride units. RB contains both a primary aromatic and a secondary aromatic amine, which differ significantly in reactivity [64]. The primary aromatic amine is more nucleophilic and is therefore expected to preferentially undergo ring-opening reactions with maleic anhydride to form stable amide/carboxylic acid functionalities, a well-established reaction pathway [65]. In contrast, the secondary aromatic amine is less reactive due to lone-pair delocalization across adjacent aromatic systems and increased steric hindrance [66]. Consequently, surface immobilization is likely to proceed predominantly through the primary aromatic amine, and modification at this site is not expected to abolish the inhibitory activity.

Both the inhibitor RB and its acetylated derivative (aRB) were analyzed for purity using NMR and ATR-IR spectroscopy.

The successful acetylation of RB was confirmed by NMR and ATR-IR analyses. The ^1^H-NMR and the ^13^C-NMR spectra, as well as the ATR-IR spectrum of aRB, are consistent with the expected molecular structure (see Figures S4–S6 in Supplementary Material). In the ATR-IR spectrum, new absorption bands corresponding to the acetyl group were observed.

### 2.3. Evaluation of the Effect of Inhibitors on HRP Activity

#### 2.3.1. Effects of RB and aRB on HRP Activity in Solution

**Figure 4:**
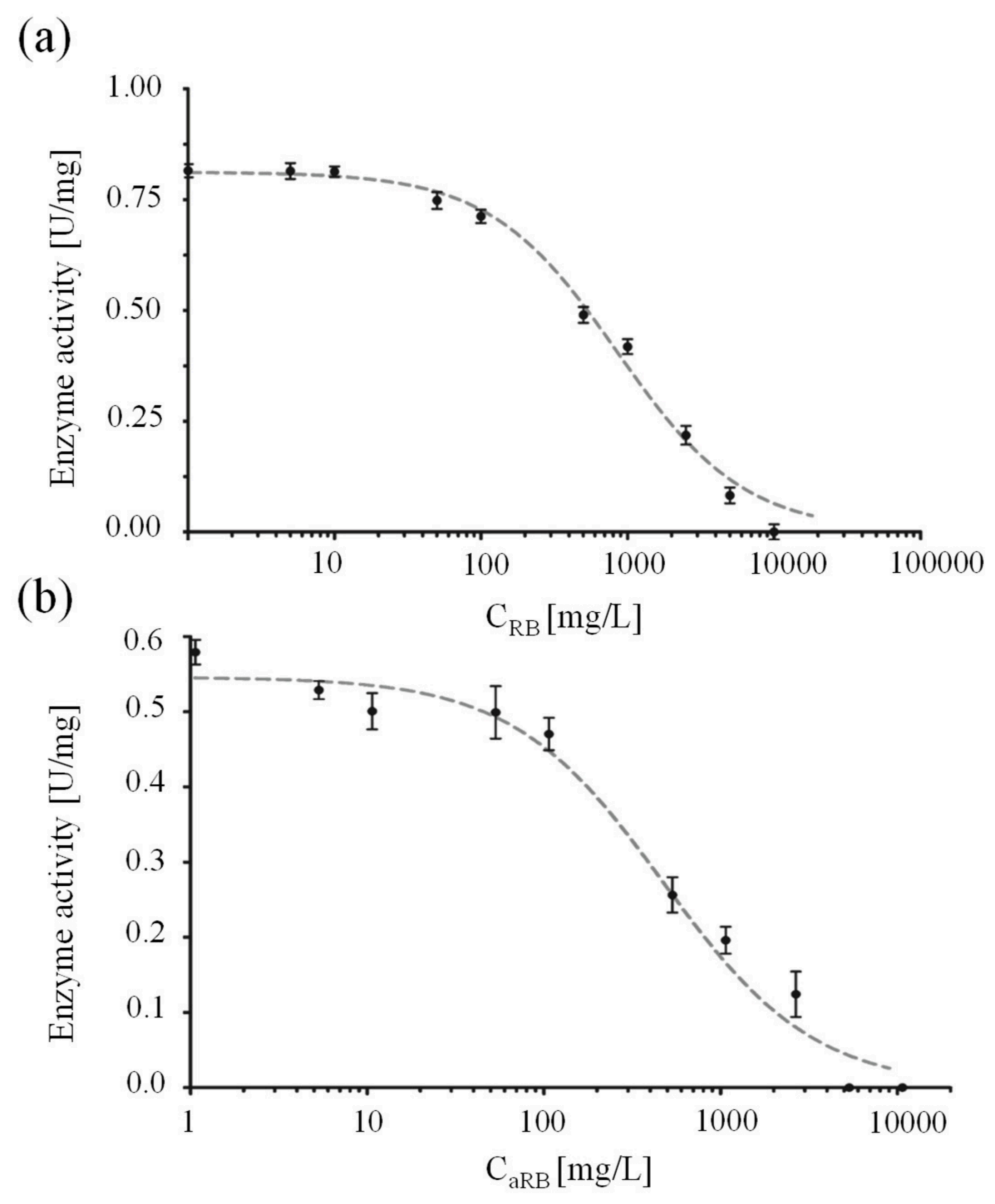
Dose–response curves for the inhibition of RB (**a**) and aRB (**b**), showing the enzyme activity *v_0_* of HRP in U∙mg^−1^ vs. logarithmically plotted concentration of RB resp. aRB, unit of c(RB) resp. c(aRB) is mg∙L^−1^, concentration of ABTS and assay composition are constant, dashed lines: four-parameter logistic (4PL) model-logIC_50_ (empirical nonlinear regression) with GraphPad Prism 5, number of replicates (*n*) = 3, (**a**) *R*^2^ = 0.9913, (**b**) *R*^2^ = 0.9837.

To determine the half-maximal inhibitory concentration (IC_50_) of RB and aRB, the HRP activity assay with 2,2′-Azino-*bis*(3-ethylbenzthiazoline-6-sulfonic acid) (ABTS) was optimized for this purpose and performed in the presence of varying inhibitor concentrations. Figure 4 shows the dose–response inhibition curves used to determine the IC_50_ values of RB (a) and aRB (b). The nonlinear regression used here represents an empirical dose–response model for IC_50_ determination and was not intended as a mechanistic inhibition analysis.

An inhibitory effect of the RB and aRB on HRP activity was observed. Using GraphPad Prism 5, an IC_50_ value of 826 mg∙L^−1^ was determined for RB, while aRB showed a lower IC_50_ value of 533 mg∙L^−1^. These results indicate that blocking the amino group by acetylation influences the inhibitory strength, with aRB exhibiting a stronger inhibitory effect on HRP than RB. Based on these findings, we conclude that binding RB to a functionalized surface is unlikely to eliminate its inhibitory effect. This gives the first hint that the primary amino group of RB can be used for surface functionalization.

Since both RB and aRB have a blue color in solution, their absorption may interfere with the absorbance measurement of ABTS. This was confirmed by mixing fully oxidized ABTS solutions with RB or aRB after reaction with HRP. The absorbance of ABTS at 405 nm was reduced by RB as well as by aRB.

#### 2.3.2. Effect of Immobilized RB on HRP Activity

**Figure 5:**
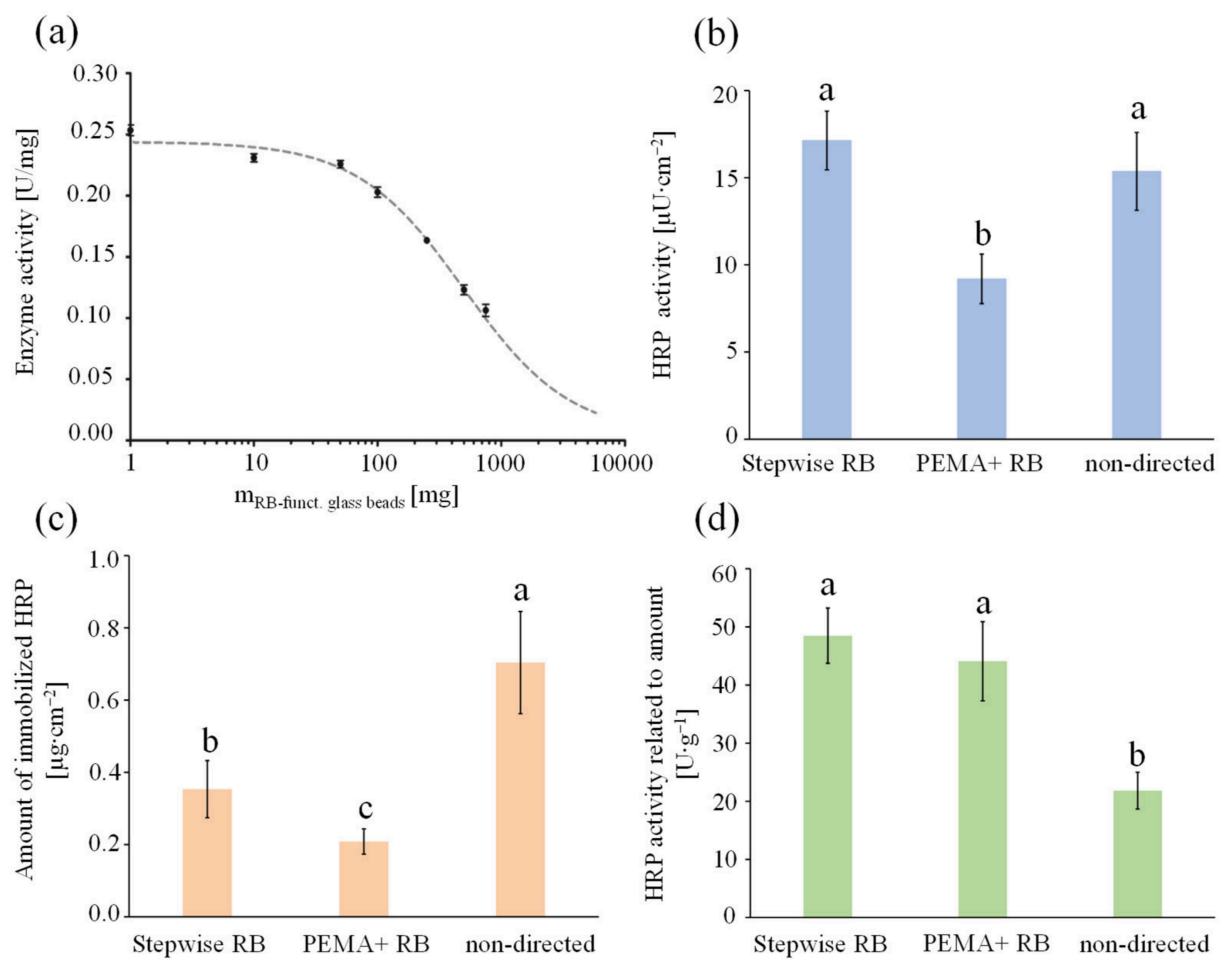
(**a**) Correlation between HRP activity and mass of RB-functionalized glass beads plotted on a logarithmic scale; dashed line: empirical 4PL logIC_50_ non-linear regression fit (GraphPad Prism 5; *n* = 3, *R*^2^ = 0.9941), used here as an apparent dose–response model rather than a mechanistic inhibition analysis. (**b**) Activity of immobilized HRP on various functionalized glass substrates. (**c**) Amount of immobilized HRP on various functionalized glass substrates. Reference values have been subtracted, (*n* = 6 for (**b**,**c**)). (**d**) Specific activity related to the amount of immobilized HRP on various glass substrates. A statistical analysis was performed for (**c**,**d**). Specifically, one-way ANOVA was applied to each dataset, followed by Tukey’s Honest Significant Difference (HSD) post hoc test for multiple comparisons (*n* = 6, α = 0.05). The results are presented significance lettering (a, b, c) above the bars; groups sharing the same letter are not significantly different (*p* > 0.05), whereas different letters indicate statistically significant differences (*p* < 0.05).

RB interferes with the ABTS-based assay, complicating direct activity measurements in solution. To evaluate the inhibitory potential of immobilized RB, an alternative experimental set-up was used. Glass beads were simultaneously functionalized with PEMA and RB and used as inhibitors in a cuvette-based system, in which the beads did not interfere with the light beam. Figure 5a shows the correlation between the amount of RB-functionalized glass beads and HRP activity.

The presence of RB-functionalized glass beads led to a reduction in HRP activity, demonstrating that immobilized RB also has an inhibitory effect on HRP. This finding further confirms that blocking the primary amino group of RB, as in aRB, does not abolish inhibition. An apparent IC_50_ value of 526 mg RB functionalized glass beads was determined.

### 2.4. Investigation of Directed Immobilized HRP

HRP immobilization was performed in a directed manner using the inhibitor-mediated, directed immobilization approach. First, cover slips were functionalized with the inhibitor RB, and HRP was applied to it. Subsequently, a second functionalized surface was brought into contact with the first, enabling covalent attachment of HRP to the second surface (Figure 1). To assess the success of directed HRP immobilization, both the enzymatic activity and the amount of immobilized HRP were analyzed.

To achieve directed, inhibitor-mediated immobilization of the enzyme, two different approaches were investigated: (a) the first surface was functionalized stepwise, first with PEMA and then with RB (stepwise RB); (b) the first surface was functionalized simultaneously with PEMA and RB (PEMA+RB). Cover slips functionalized with propylamine (PA) served as reference samples. For the second surface, PEMA-functionalized cover slips were used, while non-functionalized cover slips acted as additional controls. For comparison, random immobilization was also evaluated by covalently binding HRP directly to PEMA-functionalized cover slips. To validate the presence of the enzyme layer on the functionalized support, the substrates were analyzed by AFM and XPS (Figure S7).

AFM imaging revealed that random immobilization of HRP led to distinct changes in surface morphology (Figure S7a), characterized by the appearance of textured, globular protein features distributed across the polymer baseline. The corresponding phase imaging (Figure S7b) showed clear material contrast between the polymer layer and the attached protein features, supporting their successful deposition on the surface. XPS further confirmed the enzyme immobilization by the increase in the N 1s signal to 3.00 at. % (Table S1). The C 1s spectrum of the enzyme-modified surface (Figure S7c) was deconvoluted into distinct components: a dominant hydrocarbon (C–C/C–H) contribution at 285.2 eV, a component at 286.5 eV assigned to C–N and C–O species from the protein structure, and a higher-binding-energy component at 288.7 eV corresponding to the amide carbonyls (N–C=O) of the peptide backbone [67,68]. Additionally, a distinct feature observed at 293.2 eV is assigned to the K 2p_3/2_ signal of trace residual potassium ions from the enzymatic buffer solution [69]. Together, the AFM and XPS results are consistent with the successful immobilization of HRP on the functionalized substrate.

The activity of immobilized HRP was determined using the ABTS assay, with cover slips placed in a 6-well plate. The results of these measurements are shown in Figure 5b. A proper statistical analysis was performed for Figure 5b–d using one-way ANOVA followed by Tukey’s HSD post hoc test (*n* = 6, α = 0.05). Different letters above bars indicate statistically significant differences (*p* < 0.05).

As all tested glass substrates exhibited HRP activity, immobilization of HRP was successful for all three approaches for directed as well as non-directed HRP immobilization. The activity of immobilized HRP obtained using the PEMA+RB approach was 9.2 µU∙cm^−2^, which is lower (*p* < 0.05) than the activity measured for the stepwise RB approach (17.1 µU∙cm^−2^) and for non-directed, randomly immobilized HRP (15.4 µU∙cm^−2^). These values indicate comparable HRP activities for the stepwise RB approach and the non-directed approach, and statistical analysis confirmed that these two methods did not differ significantly (*p* = 0.239).

To determine the amount of immobilized HRP on the second surface, amino acid analysis by LC–MS/MS after complete hydrolysis was performed. The results confirmed that the contact process was successful, as significant amounts of immobilized HRP were detected. Figure 5c shows the determined amounts of immobilized HRP on the glass substrates for the different approaches. After the contact process using stepwise RB-functionalized cover slips, 0.35 µg·cm^−2^ of immobilized HRP was detected as well as 0.21 µg·cm^−2^ for the PEMA+RB approach. These values correlate with the HRP activity per cm^2^ for both surfaces directed immobilized with HRP.

The differences in activity and the amount of enzyme immobilized between the two directed approaches can be attributed to the accessibility of RB on the first surface. In the stepwise method, RB molecules are attached to an already PEMA-functionalized surface, resulting in inhibitors that are more exposed and accessible. In contrast, simultaneous functionalization with PEMA and RB leads to some RB molecules being embedded within the polymer layer, making them less accessible. This more randomized surface functionalization results in a greater proportion of hidden RB molecules, which impedes stable binding of HRP [70,71,72,73]. With the non-directed approach, the highest amount of HRP was immobilized, with a detected quantity of 0.70 µg·cm^−2^. As expected, more enzyme binds under non-directed random immobilization [19,35]. The use of inhibitors in inhibitor-mediated immobilization leads to a reduction in the amount of enzyme at the functionalized surface as a limited amount of inhibitor is available. Figure 5b,c show that the amount of bound HRP generally correlates with the measured HRP activities on the functionalized cover slips, except in the case of the stepwise approach. Here, the overall measured activity is comparable to that of the non-directed approach, even though a significantly lower amount of protein was detected. This suggests that the orientation of HRP molecules plays a crucial role in determining HRP activity [23]. In the non-directed approach, a larger amount of HRP is immobilized (Figure 5c, *p* < 0.0001); however, the corresponding activity is comparatively low, indicating that a substantial portion of the enzyme is inactive or inaccessible. By relating the measured HRP activities to the amounts of immobilized enzyme, the following values for specific enzyme activity were obtained: 0.048 U·mg^−1^ for stepwise RB (4.4% of HRP activity in solution), 0.044 U·mg^−1^ for PEMA+RB (4.05%), and 0.022 U·mg^−1^ for non-directed immobilization (2.02%) (Figure 5d). These data demonstrate that the directed contact approaches yield approximately twofold higher specific activities compared to non-directed immobilization. Statistical analysis confirmed a significant difference in specific activity between the directed and non-directed approaches, with no significant difference observed between stepwise RB and PEMA+RB (*p* = 0.326). The higher specific activity observed for the directed approaches is consistent with oriented strategies that preserve active-site accessibility by minimizing unfavorable orientations and conformational distortion [27,28]. This interpretation of oriented binding can be further supported by literature-based estimates of the HRP–RB interaction. RB has been reported to act as a strong competitive inhibitor of HRP at pH values above six [48], exhibiting an affinity significantly higher than that of common substrates, with reported *K*_M_/*K*_i_ ratios of approximately 150 for ABTS and 350 for phenol [48]. Considering that typical *K*_M_ values for HRP substrates lie in the low-millimolar range (e.g., ~2.0 mM for ABTS at pH 7.0 [74] and ~2.3 mM for phenolic compounds such as 2,4-dichlorophenol at pH 6.5 [75], these ratios suggest that *K*_i_ (which approximates the dissociation constant, *K*_d_, in a competitive model) is in the low micromolar range. This indicates a relatively strong but reversible interaction between HRP and RB, which could promote preferential docking of the enzyme via its active site onto the RB-functionalized PEMA surface. Reported association rate constants for HRP–aromatic ligand interactions typically fall within the range of *k*_on_ ≈ 10^4^–10^8^ M^−1^ s^−1^ [76,77]. Assuming a dissociation constant in the micromolar range, this corresponds to a kinetic regime of rapid-to-moderate exchange, with binding lifetimes on the millisecond-to-second timescale. These estimates should be interpreted as order-of-magnitude approximations, as they are derived from literature data obtained under varying experimental conditions.

Such a kinetic regime is consistent with the proposed immobilization mechanism: RB–HRP interactions are expected to be sufficiently strong to enable preferential active-site docking, while remaining reversible enough to allow dynamic exchange prior to covalent attachment during the contact process. This interpretation is further supported by structural insights; for example, Veitch et al. [78] highlight the flexibility of the HRP aromatic-binding channel, which accommodates bulky ligands through interactions with distal residues such as Arg38. Furthermore, studies on HRP in confined or surface-associated environments demonstrate that local interactions can influence substrate affinity and catalytic efficiency [79]. These findings are consistent with our observation of enhanced specific activity, suggesting that RB-mediated docking promotes a favorable enzyme orientation.

The physical basis for this oriented docking can be further understood by considering the three-dimensional structure of HRP and its surface-exposed conjugation sites. HRP is a globular, heme-containing glycoprotein in which the catalytic heme pocket and surrounding residues define the substrate- and inhibitor-binding region [80]. The active site features a hydrophobic, aromatic-binding pocket containing key distal residues, notably Arg38 and His42, which stabilize ligands through hydrogen bonding and aromatic interactions, while His170 coordinates the central heme iron [80,81]. As a competitive inhibitor, RB is expected to bind within or adjacent to this aromatic region, thereby orienting the enzyme with its active site toward the RB-functionalized support. Covalent immobilization can then occur through the reaction of the PEMA maleic anhydride groups with surface-exposed primary amines on HRP, particularly the ϵ-amino groups of lysine residues such as Lys174 and Lys232, as well as the N-terminal amine [53,82]. Because these reactive sites are located on protein surfaces distant from the catalytic pocket, this structural arrangement is expected to spatially separate the docking and anchoring sites. This spatial control effectively minimizes random attachment, preserving excellent substrate access to the active site and driving the observed twofold increase in specific activity.

To assess the decrease in activity caused by immobilization, the initial activity of HRP in solution was determined to be 1.087 ± 0.024 U·mg^−1^. All immobilized HRP samples exhibited substantially lower activity compared to free HRP in solution, with activities approximately 23-, 25-, and 49-fold lower for the stepwise RB, PEMA+RB, and non-directed immobilization approaches, respectively. The detailed quantitative parameters and catalytic performance of the free and immobilized HRP systems are summarized in Supplementary Table S2.

This reduction is consistent with the commonly observed decrease in enzymatic activity following immobilization and can be attributed to mass-transfer limitations, microenvironmental changes around the enzyme, the presence of random immobilization, and structural or steric constraints imposed by the support [83,84,85,86,87].

Our findings align with literature reports on site-directed immobilization strategies. For example, site-directed subtilisin immobilization via single-point cysteine attachment achieved 28–83% of free enzyme activity and nearly 3-fold higher activity than random wild-type immobilization (11%) on the same membrane [35]. Similarly, ligand-directed HRP immobilization using Langmuir–Blodgett films achieved a specific activity of 182.1 U·mg^−1^ (4.5-fold higher than random adsorption at 40.5 U·mg^−1^). However, both values remained substantially lower than that of the free enzyme (2250 U·mg^−1^) [83]. Enhanced catalytic performance through controlled orientation has also been demonstrated for other systems, as shown by reported site-directed subtilisin immobilization yielding more than a sevenfold increase in catalytic efficiency relative to random attachment [36]. In addition, site-directed immobilization of L-HAD@AmR increased catalytic efficiency threefold relative to the free enzyme and maintained 81% activity after six cycles, whereas random adsorption retained only 22% after three cycles [37]. Furthermore, lipase immobilized on polyphenol-modified magnetic nanoparticles using computational lysine selection recovered 71.3% activity (1.7-fold higher than random) and achieved superior biodiesel yield [40]. Collectively, these studies confirm that oriented immobilization strategies enhance activity relative to non-directed attachment, supporting the observations reported here.

In summary, we present a robust strategy for site-directed enzyme immobilization using immobilized competitive inhibitors. This approach not only doubles the specific activity compared to non-directed immobilization but also preserves the native enzyme, requiring no molecular modifications, offering a simple yet effective platform for efficient biocatalysis.

## 3. Materials and Methods

### 3.1. Chemicals

All chemicals were obtained from commercial sources and were used as received. Ultrapure water (UPW) was produced in the laboratory using a purelab flex purification system from ELGA LabWater (Veolia Water Technologies Deutschland GmbH, Celle, Germany). Peroxidase from Horseradish (~150 U/mg), Remazol Brilliant Blue R, Poly(ethylene-*alt*-maleic anhydride) (average MW 100,000–500,000, powder), Propylamine (≥99%), Amino acid (analytical standard), *N*-Acetyl-*L*-glutamine (≥99%) and *S*-(2-Aminoethyl)-*L*-cysteine hydrochloride (≥98%) were purchased from Sigma Aldrich (St. Louis, MO, USA). All other chemicals were of analytical grade.

### 3.2. Analysis

Attenuated total reflection Fourier-transform infrared (ATR-FT-IR) spectra were recorded from 4000–450 cm^−1^ using a Nicolet iS10 FT-IR spectrometer (Thermo Fisher Scientific Inc., Waltham, MA, USA) with a diamond ATR crystal at room temperature (32 scans, 1 cm^−1^ resolution). Spectra were processed using OMNIC Spectra software 2.2. LC–MS/MS analyses were performed on an Agilent 6420 Triple Quadrupole LC/MS system (Agilent Technologies Inc., Santa Clara, CA, USA). Amino acid analysis was conducted using a Luna 5 µm SCX 100 Å column (150 × 2 mm) with a guard cartridge (4 × 2 mm) (Phenomenex Inc., Torrance, CA, USA). NMR spectra (^1^H NMR 400 MHz; ^13^C NMR 101 MHz) were recorded on a Bruker Avance Neo 400 spectrometer (Bruker Corp., Billerica, MA, USA). Data were analyzed using MNova software 11.0 (Mestrelab Research, S.L., Santiago de Compostela, Spain). Deuterated solvents (CDCl_3_ and DMSO-*d*_6_) were used. Chemical shifts are reported in ppm; multiplicities were abbreviated as s—singulet, d—doublet, t—triplet, sept—septet, m—multiplet, br—broad.

### 3.3. Methods

#### 3.3.1. ABTS Assay for HRP Activity

To determine the enzymatic activity of HRP, the ABTS assay was employed, in which ABTS serves as the substrate. The procedure for the assay performed in a microplate reader was adapted from Siguemoto et al. [88]. 125 µL of 67 mM phosphate buffer (pH 7.0), 17 µL of a 1.7 mM ABTS solution, and 17 µL of a 0.8 mM hydrogen peroxide solution were added to a microplate well of a 96-well plate. After addition of 42 µL of an HRP solution, the absorbance at 405 nm was monitored for at least 5 min in a kinetic assay. The assay was carried out at room temperature. The absorbance at 405 nm was plotted as a function of time (in minutes), and the slope was calculated. According to the Beer–Lambert law, HRP activity can be calculated using the molar extinction coefficient of ABTS (31,100 L∙mol^−1^∙cm^−1^). One unit of enzymatic activity (U) is defined as the amount of enzyme that catalyzes the oxidation of 1 µmol of ABTS per minute under standard assay conditions.

To investigate the influence of the inhibitor RB, 100 µL of an RB solution in UPW (concentration range: 1–10,000 mg·L^−1^) was added, followed by the addition of the HRP solution. When acetylated RB was used, 100 µL of an aRB solution in UPW (concentration range: 1–10,670 mg·L^−1^) was added accordingly.

The ABTS assay was also applied to determine the enzymatic activity of HRP immobilized on functionalized glass beads, using quartz glass cuvettes instead of microtiter plates. First, the appropriate amount of functionalized glass beads was placed in a quartz glass cuvette together with 997 µL of 67 mM potassium phosphate buffer (pH 7.0). The suspension was incubated for 5 min at room temperature on an orbital shaker at 120 rpm. Subsequently, 136 µL of ABTS solution (0.17 mM in phosphate buffer) and 136 µL of a 0.8 mM hydrogen peroxide solution (in phosphate buffer) were added. After mixing, 335 µL of HRP solution was added, and the kinetic measurement at 405 nm was started and recorded for at least 5 min.

The ABTS assay was further used to determine the activity of immobilized HRP on functionalized cover slips. The assay was performed in 6-well plates in which the functionalized cover slips were positioned. Therefore, 1994 µL of 67 mM potassium phosphate buffer (pH 7.0), 272 µL of ABTS solution (1.7 mM in phosphate buffer), and 272 µL of a 0.8 mM hydrogen peroxide solution (in phosphate buffer) were added. The absorbance at 405 nm was monitored for at least 30 min.

#### 3.3.2. Synthesis and Characterization of Acetylated RB

Acetylation of RB was performed to investigate the influence of the primary amino group on the inhibitory effect of RB on HRP. Therefore, 1 mmol of RB was placed in a 25 mL two-necked flask equipped with a magnetic stirrer and a reflux condenser and cooled in an ice bath. Subsequently, 250 µL of a 4 M sodium hydroxide solution and 10 mL of UPW were added. While stirring and cooling with ice water, 2 mmol of acetyl chloride was added dropwise, and the reaction mixture was stirred in the ice bath for 30 min. After transferring the solution to a 50 mL round-bottom flask, the mixture was evaporated to dryness using a rotary evaporator. The residue was dried overnight in a drying oven at 100 °C. A dark purple solid was obtained with a yield of 95.1%.

**Figure 6:**
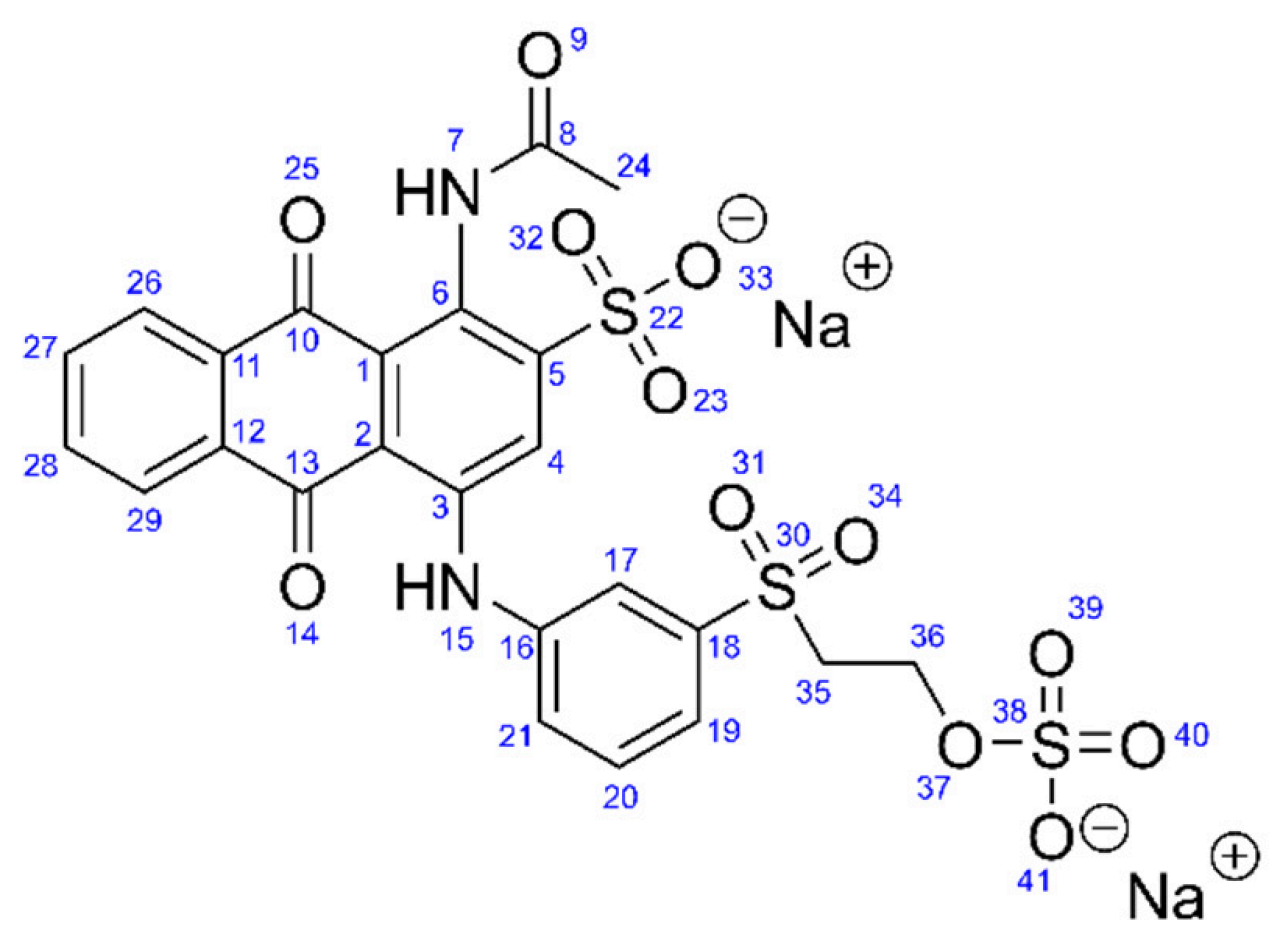
Structural formula of aRB with atom numbering.

For NMR analysis, aRB was dissolved in DMSO-*d*_6_. The numbering of the structural formula used for NMR analysis differs from the IUPAC nomenclature (see Figure 6). Proton assignments refer to the numbering of the corresponding carbon or nitrogen atoms. The characteristic values were given as follows: shift in ppm (multiplicity, integral, assignment to the corresponding carbon or hydrogen atoms).

^1^H-NMR (400 MHz, DMSO-*d*_6_): δ 11.82 (s, 1H, H-15, -NH), 10.03 (s br, 1H, H-7, -NH acetylated), 8.26 (m, 2H, Ar-H), 8.04 (s, 1H, Ar-H), 7.86 (m, 3H, Ar-H), 7.75 (s, 1H, Ar-H), 7.65–7.60 (m, 2H, Ar-H), 3.99 (t, 2H, H-36, methylene group connected to sulfate group), 3.68 (t, 2H, H-35, methylene group connected to sulfonyl group), 3.36 (water peak of DMSO), 2.49 (DMSO-*d*_6_), 2.51 (s, theoretical 3H, H-24, methyl group of acetyl group). (see Figure S4 in the Supplementary Material).

^13^C-NMR (101 MHz, DMSO-*d*_6_): δ 183.27 (s, 1C, C-10), 182.22 (s, 1C, C-13), 169.30 (s, 1C, C-8), 144.67 (s, 1C, C-16), 142.17 (s, 1C, C-18), 141.00 (s, 1C, C-3), 140.81 (s, 1C, C-5), 138.56 (s, 1C, C-11), 134.12 (s, 1C, C-12), 133.59 (s, 1C, C-28), 133.42 (s, 1C, C-27), 133.05 (s, 1C, C-6), 130.66 (s, 1C, C-20), 126.52 (s, 1C, C-21), 126.13 (s, 2C, C-29, C-26), 123.97 (s, 1C, C-4), 122.35 (s, 1C, C-2), 120.84 (s, 1C, C-1), 113.36 (s, 1C, C-17), 109.65 (s, 1C, C-19), 59.07 (s, 1C, C-35), 54.66 (s, 1C, C-36), 39.51 (sept, DMSO-*d*_6_), 24.05 (s, 1C, C-24). (see Figure S5 in the Supplementary Material).

ATR-IR (ν_max_/cm^−1^): 3420 (-NH stretching), 3066 (=C-H stretching), 2923 (-CH_2_ stretching), 1713 (-C=O stretching), 1634 (-C=O stretching amide), 1582 (-NH bending amide), 1495 (aromatic ring), 1353, 1258 (-CH_2_ bending), 1220, 1167 (-SO_2_ stretching), 1137, 1023 (S=O stretching), 936, 882, 754, 729 (=C-H bending) (see Figure S6 in the Supplementary Materials).

#### 3.3.3. Surface Functionalization of Glass Materials with Copolymer PEMA

Glass cover slips (24 × 24 mm^2^) and glass beads (ø 0.5 mm) were used for the functionalization. The cover slips were placed in wafer baskets to facilitate handling. The procedure was based on the method described by Pompe et al., with minor modifications [50]. First, the glass materials were cleaned. They were sonicated in an ultrasonic bath for 30 min each with UPW, EtOH and subsequently three additional times with UPW. The volume of each washing solution was 250 mL for the cover slips and 70 mL for the glass beads. After pre-cleaning, the glass materials were subjected to oxidative pretreatment using an RCA solution. This procedure is based on a cleaning process developed by the Radio Corporation of America. A mixture of H_2_O_2_, ammonia, and UPW in a ratio of 1:1:5 was heated to 70 °C in a quartz glass beaker, and the glass substrates were immersed for 10 min. Afterwards, the glass substrates were rinsed thoroughly three times with UPW and then dried in a drying oven.

For aminosilanization, a 20 mM solution of APTES in 90% (*v*/*v*) isopropanol was prepared. During the experiment, 250 mL of the solution was used for the cover slips and 50 mL for the glass beads. The cooled glass substrates were incubated in this solution for 2 h under a nitrogen atmosphere in a large desiccator while being gently agitated on an orbital shaker at approximately ~50 rpm. After incubation, the glass materials were rinsed thoroughly three times with isopropanol and subsequently dried at 120 °C for at least 1 h.

Functionalization with PEMA was performed using a 0.5–2.5% (*w*/*w*) PEMA solution in a mixture of tetrahydrofuran and acetone (2:1, *v*/*v*). For the cover slips stored in teflon basket, 250 mL of the solution was used, while 50 mL was used for the glass beads stored in screw-cap glass bottles. For better mixing, the glass containers were placed on an orbital shaker at ~150 rpm. After cooling to room temperature, the glass substrates were immersed in the PEMA solution for 24 h. Subsequently, the glass materials were immediately tempered in a drying oven at 120 °C for 2 h.

After cooling to room temperature, the glass materials were rinsed twice with acetone for 15 min each, using 250 mL of acetone for the cover slips and 50 mL for the glass beads. The glass substrates were then dried under a stream of nitrogen and tempered at 120 °C for 2 h. Prior to further use, the heat treatment was repeated.

#### 3.3.4. Surface Functionalization of PEMA-Functionalized Cover Slips with RB

PEMA-functionalized cover slips were stepwise functionalized with RB. The cover slips were fixed in home-built immobilization chambers. For functionalization with RB, 500 µL of a 10 mg∙mL^−1^ RB solution in UPW was added to each cover slip. The chambers were then sealed, and the cover slips were incubated for 24 h at room temperature. For agitation, the chambers were placed on an orbital shaker at 80 rpm. After incubation, the RB solution was carefully removed, and the cover slips were rinsed four times with 1 mL of UPW for 10 to 15 min each. Afterwards, the cover slips were dried under a stream of nitrogen. Until further use, the RB-functionalized cover slips were stored in 6-well plates with the functionalized side facing upward.

As reference samples, PEMA-functionalized cover slips were functionalized with PA instead of RB. Since PA, unlike RB, is a concentrated liquid, only 300 µL of PA was used for functionalization. Apart from this difference, the procedure was identical.

#### 3.3.5. Simultaneous Surface Functionalization of Glass Substrates with PEMA and RB

Glass beads and cover slips were prepared and aminosilanized as described in Section 3.3.3. The functionalization solution was prepared by dissolving 2% (*w*/*w*) PEMA and 1% (*w*/*w*) RB in a mixture of acetone and DMSO in a 2:1 (*v*/*v*) ratio. To ensure complete dissolution, PEMA was first dissolved in the appropriate volume of acetone. Subsequently, a small amount of DMSO was added, followed by RB. The mixture was shaken thoroughly and then adjusted to the final volume with the remaining amount of DMSO. The solution was transferred to a screw-cap glass bottle and agitated on an orbital shaker for 30 min at 120 rpm.

The glass materials were added to the PEMA-RB-solution and incubated on an orbital shaker at 150 rpm for 20–24 h. Subsequently, the glass beads were tempered in a drying oven at 120 °C for 2 h, followed by rinsing of the glass materials three times with DMSO (50 mL each) for 15 min on an orbital shaker at 120 rpm. During this step, the solution turned blue due to dissolution of unbound RB. The glass materials were then rinsed three times with 50 mL of acetone (15 min each at 120 rpm) to remove unbound PEMA.

After three additional washing steps with DMSO and three with acetone, the glass beads were again tempered in a drying oven at 120 °C for at least 2h. After drying, light blue glass beads were obtained.

In order to avoid the presence of free anhydride groups, the functionalized cover slips were incubated in a 1% (*w*/*w*) RB solution in DMSO for 4 h at 100 rpm. Afterwards, the cover slips were rinsed three times with 25 mL of DMSO for 15 min each on an orbital shaker at 120 rpm and subsequently dried in a drying oven at 120 °C for at least 2 h. Light blue glass substrates were obtained.

As reference samples, cover slips were simultaneously functionalized with PEMA and PA, i.e., PA was used instead of RB. The aminosilanized cover slips were incubated in a solution containing 2% (*w*/*w*) PEMA and 1% (*w*/*w*) PA in a mixture of THF and acetone (2:1, *v*/*v*). The procedure was almost identical to that used for simultaneous functionalization with PEMA and RB, except that only three rinsing steps with 50 mL of acetone were required. To block remaining free anhydride moieties, the cover slips were incubated in a 1% (*v*/*v*) PA solution in acetone and rinsed afterwards with acetone.

#### 3.3.6. Immobilization of HRP and Contact Process

Self-constructed immobilization chambers were used. The procedure was based on that described by Cordeiro et al., with minor modifications [51]. After tempering the cover slips at 120 °C for 2 h, they were fixed in the immobilization chamber. Subsequently, 300 µL of a 1 mg∙mL^−1^ HRP solution in potassium phosphate buffer was added, and incubation was carried out on an orbital shaker at 120 rpm for 24 h. Afterwards, the HRP solution was carefully removed, and each cover slip was washed five times with 1000 µL of potassium phosphate buffer for 10 min on the orbital shaker at 120 rpm.

For the contact process, tempered PEMA-functionalized cover slips were wetted with 50 µL of the buffer and covered with the moist HRP-functionalized cover slips, with the enzyme-facing side oriented toward the PEMA. Care was taken to ensure that no air bubbles were present between the layers. By carefully screwing the lid of the chamber, both cover slips were brought into contact, and excess buffer solution was pressed out from between the layers. After an incubation time of 24 h, the cover slips were carefully separated by sliding them against each other. Subsequently, the cover slips were rinsed thoroughly with UPW, dried under a nitrogen stream, and stored in 6-well plates (functionalized side up) until further use.

#### 3.3.7. Total Hydrolysis of Proteins

To quantify proteins by amino acid analysis, the proteins must first be completely hydrolyzed. Acidic gas phase hydrolysis with hydrochloric acid was used for this purpose. First, the hydrolysis chamber was cleaned with UPW in an ultrasonic bath for 10 min, and all glassware used (including the hydrolysis vials) was cleaned using RCA solution (see Section 3.3.3.).

Cover slips or liquid samples were placed in the hydrolysis vials. The vials were then transferred into the hydrolysis chambers using clean tweezers. The chamber was sealed, and the samples were dried under vacuum for 1 h and then gassed with nitrogen. Subsequently, 4 mL of 6 M hydrochloric acid containing 1% (*v*/*v*) phenol was added to the hydrolysis chamber. After resealing the chamber, the following procedure was repeated three times: evacuation for 15 s, followed by gassing with nitrogen for 12 s. The chamber was then fully evacuated and incubated at 110 °C in a drying oven for 21 h.

After incubation, the sample vials were removed from the chamber and rinsed externally with ethanol. The hydrochloric acid was discarded, and the chamber was rinsed with ethanol. The vials were returned to the chamber and dried under a vacuum. Subsequently, the chamber was vented with nitrogen, and 30 µL of a basic solution (UPW: ethanol: trimethylamine, 2:2:1) was added to each sample for neutralization. The samples were dried again under vacuum until no liquid remained, sealed with Parafilm, and stored at −18 °C.

#### 3.3.8. Amino Acid Analysis by LC-MS/MS

After the samples were subjected to complete acidic hydrolysis, 200 µL of 30 mM ammonium acetate (AA) buffer (pH 6.0) was added to each sample vial, and the samples were rinsed 100 times with this solution. The resulting solution was transferred to reaction tubes and centrifuged at 12,000 rpm for 5 min. From the supernatant, 150 µL was transferred into an HPLC vial and supplemented with 50 µL of a 10 µM internal standards (ISTD) solution. For the ISTD solution, *N*-acetyl-*L*-glutamine (ISTD 1) and *S*-(2-aminoethyl)-*L*-cysteine hydrochloride (ISTD 2) were dissolved in AA buffer, resulting in a final concentration of 2.5 µM ISTDs in the sample solutions.

Amino acid analysis via LC-MS/MS was performed following a method adapted from the work of Thiele et al. [89]. For liquid chromatography, AA buffer was used as solvent A, and 5% (*v*/*v*) acetic acid (HAc) was used as solvent B. Both solvents were prepared with HPLC-grade chemicals and filtered through a 0.45 µm cellulose acetate membrane. The Luna 5 µm SCX 100 Å column (150 × 2 mm, with a security cartridge screwed on top, 4 × 2 mm, Phenomenex) was conditioned with 150 mM AA solution at 0.2 mL∙min^−1^, followed by flushing with a mixture of AA buffer and HAc (12.5:87.5) for 4 h.

The LC-MS/MS system combines high-performance liquid chromatography (LC) with a triple quadrupole (MS/MS) detection system. The LC system includes a solvent degasser, a binary pump, an autosampler with thermostat (set to 25 °C), a thermostatted column oven, and a variable-wavelength detector (VWD). The mass spectrometer operated in positive ESI mode, using a capillary voltage of 4000 V and a nebulization pressure of 15 psi for nebulizing the sample solution separated by HPLC. Nitrogen was used as the drying gas at 300 °C. The amino acid analysis was performed in multiple-reaction-monitoring (MRM) mode. The temperature of the column oven was 40 °C and the analysis was done with a time program that adjusts the composition of the solvents. The method is different from that described by Thiele et al. [89]. Initially, the flow rate was 0.5 mL∙min^−1^ with the solvent composition of 5% AA buffer (A) and 95% HAc (B). After 14 min, the flow rate was reduced to 0.4 mL∙min^−1^, and the solvent composition was changed to 70% A and 30% B within 2 min. After holding for 18 min, the composition of the solvent was returned to the initial conditions (5% A, 95% B, flow rate 0.5 mL∙min^−1^) within 2 min and maintained for 4 min. The total run time was 40 min, followed by a 15 min post-run under the initial conditions.

LC-MS/MS control and data acquisition were performed using MassHunter B.08.00 Data Acquisition software, whereas data quality was assessed with MassHunter Qualitative Analysis, and data quantification was carried out with MassHunter Quantitative Analysis. For calibration, an amino acid standard was used in a concentration range between 0.075 and 75 µM in AA buffer including the ISTDs with a concentration of 2.5 µM.

## 4. Conclusions

We developed an oriented enzyme immobilization strategy that circumvents the need for protein modification by exploiting the specific interaction between an enzyme and its inhibitor.

By investigating the inhibitory effect of an acetylated derivative of RB on HRP, we demonstrated that the primary amino group of RB can be used for immobilization. The use of RB functionalized glass beads in the enzyme activity assay showed a clear inhibition of HRP, confirming that immobilized RB has an inhibitory effect on HRP. RB was therefore used as an inhibitor to enable directed, inhibitor-mediated immobilization of HRP. The active immobilized HRP displayed approximately twofold higher specific activity than that obtained by non-directed immobilization, successfully proving our target-directed approach. While this study establishes a fundamental proof-of-concept for inhibitor-mediated site-directed immobilization, it also opens avenues for adapting the choice of ligand to expand this contact process to a wider range of target enzymes. Given the enhanced specific activity demonstrated here, this methodology serves as a promising foundation for developing more efficient heterogeneous biocatalysts in specialized applications such as biosensors or array-based systems. To further assess the industrial relevance of this approach, future work should focus on the comprehensive physicochemical and kinetic profiling of these immobilized biocatalysts to systematically evaluate their thermal, pH, and operational stability.

## Data Availability

The original contributions presented in this study are included in the article/Supplementary Materials. Further inquiries can be directed at the corresponding author.

## Supplementary Materials

The following supporting information can be downloaded at: https://www.mdpi.com/article/10.3390/molecules31152669/s1, Figure S1: ^1^H-NMR (400 MHz) of RB in DMSO-*d*_6_; Figure S2: ^13^C-NMR (101 MHz) of RB in DMSO-*d*_6_; Figure S3: ATR-IR of RB; Table S1. Surface elemental composition (at. %) of the functionalized glass substrates at each modification step; Figure S4: ^1^H-NMR (400 MHz) of acetylated RB in DMSO-*d*_6_; Figure S5: ^13^C-NMR (101 MHz) of acetylated RB in DMSO-*d*_6_; Figure S6: ATR-IR of acetylated RB; Figure S7: AFM images of (a) the HRP-immobilized surface, along with (b) the corresponding phase image; (c) high-resolution XPS C 1s spectrum of HRP; Table S2. Quantitative immobilization parameters and catalytic performance of the free HRP and immobilized HRP systems.
