# Supplementary material for: Directed Immobilization of Horseradish Peroxidase Using a Covalently Attached Competitive Inhibitor
Source: Molecules. 2026 Jul 31;31(15):2669. doi: 10.3390/molecules31152669 (PMC13468518; doi:10.3390/molecules31152669)
Supplement: Supplementary file 1 [file molecules-31-02669-s001.zip › molecules-4195699-supplementary.pdf]

Supporting information

Directed Immobilization of Horseradish Peroxidase Using a Covalently Attached Competitive Inhibitor

Anne Muschter [1,2], Sophia Rosencrantz [1,3], Takwa Chouki [1,2], Stefan Reinicke [1,2], and Ruben R. Rosencrantz [1,2]*

1 Fraunhofer Institute for Applied Polymer Research IAP; Division of Life Science & Bioprocesses, Geiselbergstr. 69, 14476 Potsdam, Germany
2 Brandenburg University of Technology (BTU) Cottbus-Senftenberg, Chair for Biofunctional Polymer Materials, Universitätsplatz 1, 01968 Senftenberg, Germany
3 Fraunhofer Cluster of Excellence Immune-Mediated Diseases CIMD, Frankfurt am Main, Germany

* Correspondence: ruben.rosencrantz@iap.fraunhofer.de

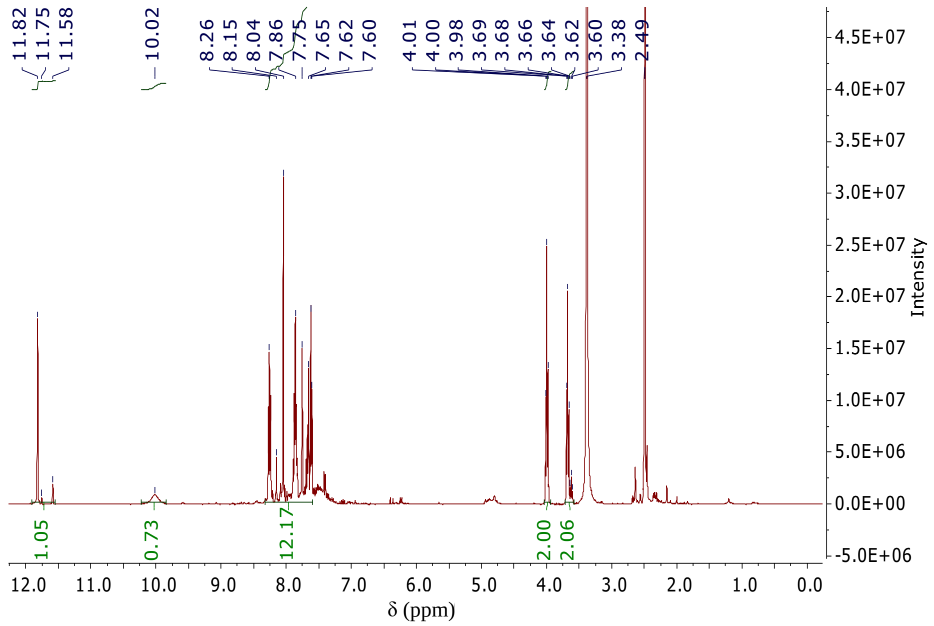

^{1}H NMR (400 MHz, DMSO-d_6): δ = 11.82 (s, 1H, H-7), 10.02 (s, 1H, H-19), 8.26 (m, 2H, Ar-H), 8.04 (s, 1H, Ar-H), 7.86 (m, 3H, Ar-H), 7.75 (s, 1H, Ar-H), 7.65-7.60 (m, 2H, Ar-H), 4.00 (t, 2H, H-33), 3.68 (t, 2H, H-32), 3.38 (water peak of DMSO), 2.49 (DMSO-d_6).

Figure S1. ^{1}H-NMR (400 MHz) of Remazol Blue in DMSO-d_6

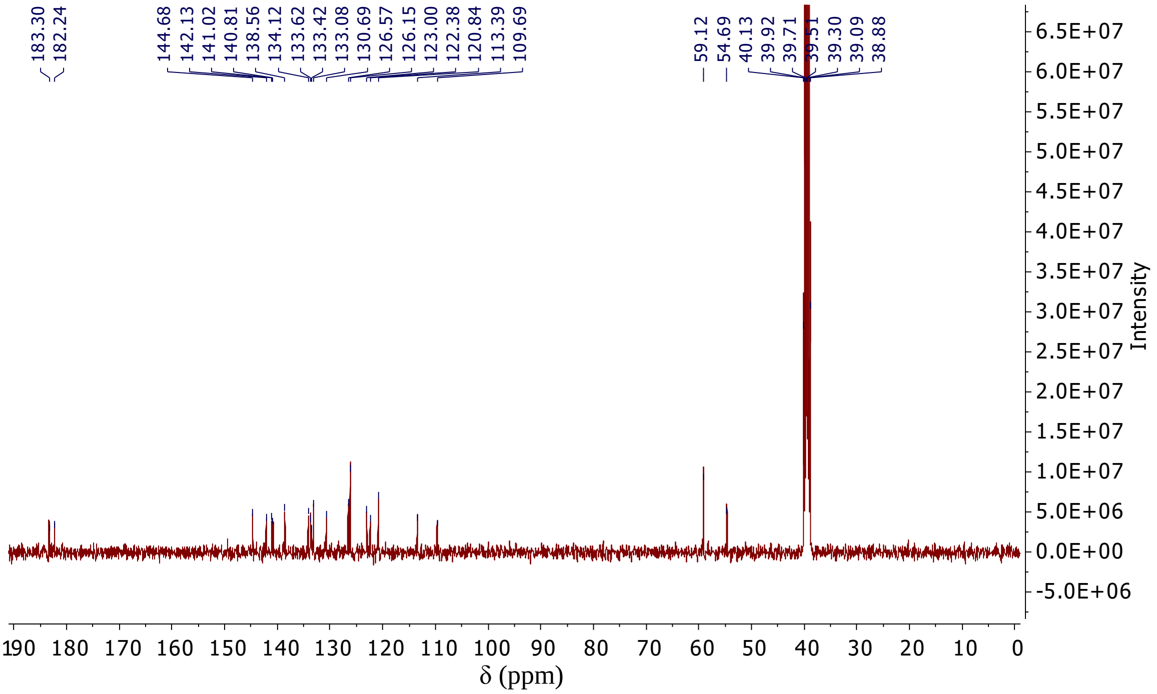

^{13}C NMR (101 MHz, DMSO d_6): δ = 183.30 (s, 1C, C-17); 182.24 (s, 1C, C-14); 144.68 (s, 1C, C-8); 142.13 (s, 1C, C-10); 141.02 (s, 1C, C-15); 140.81 (s, 1C, C-16); 138.56 (s, 1C, C-3); 134.12 (s, 1C, C-6); 133.62 (s, 1C, C-26); 133.42 (s, 1C, C-27); 133.08 (s, 1C, C-4); 130.69 (s, 1C, C-12); 126.57 (s, 1C, C-13); 126.15 (s, 2C, C-25, C-28); 123.00 (s, 1C, C-5); 122.38 (s, 1C, C-1); 120.84 (s, 1C, C-9); 113.39 (s, 1C, C-11); 109.69 (s, 1C, C-2); 59.12 (s, 1C, C-32); 54.69 (s, 1C, C-33); 39.51 (sept, DMSO d_6).

Figure S2. ^{13}C-NMR (101 MHz) of Remazol Blue in DMSO-d_6

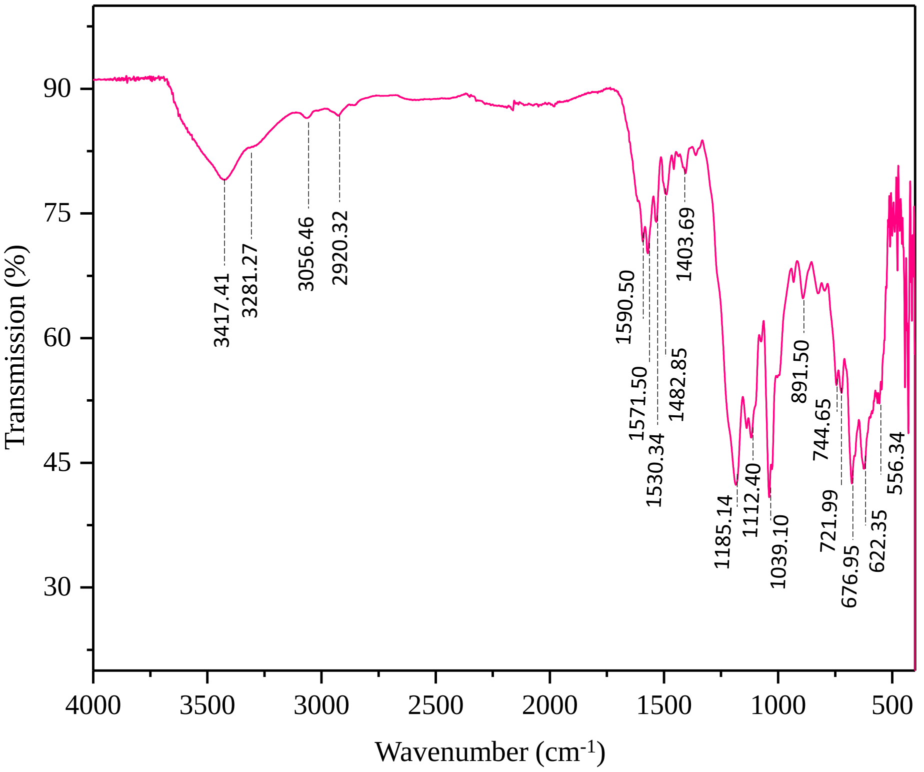

Figure S3. ATR-IR of Remazol Blue

Table S1. Surface elemental composition (at. %) of the functionalized glass substrates at each modification step:

Samples	C 1s (%)	N 1s (%)	O 1s (%)	Si 2p (%)	Cl 2p (%)	K 2p (%)
APTES	48.44	6.84	21.72	15.73	7.18	0.10
PEMA	66.33	0.03	33.11	0.52	n.d.	n.d.
HRP	60.28	3.00	31.14	3.91	1.35	0.32

n.d.: not detected

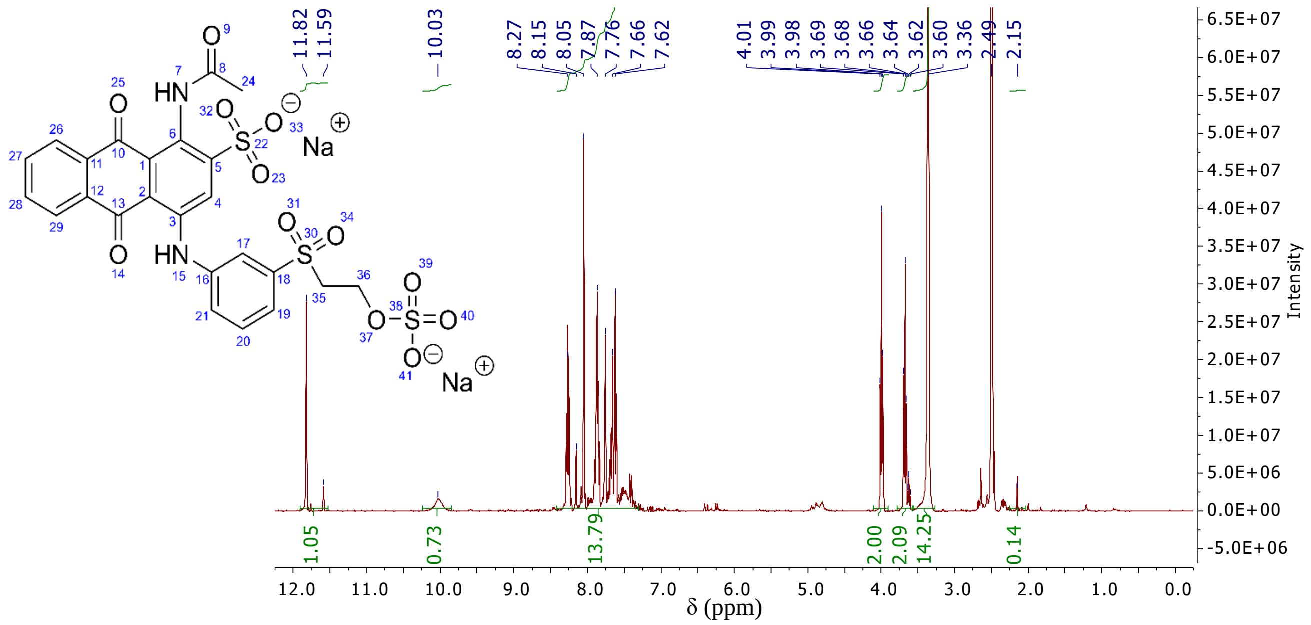

^{1}H NMR (400 MHz, DMSO-d_6): δ = 11.82 (s, 1H, H-15); 10.03 (s br, 1H, H-7); 8.26 (m, 2H, Ar-H), 8.04 (s, 1H, Ar-H), 7.86 (m, 3H, Ar-H), 7.75 (s, 1H, Ar-H), 7.65-7.60 (m, 2H, Ar-H); 3.99 (t, 2H, H-36); 3.68 (t, 2H, H-35); 3.36 (s, DMSO Water peak); 2.49 (DMSO); 2.51 (s, 3H, H-24).

Figure S4. ^{1}H-NMR (400 MHz) of acetylated Remazol Blue in DMSO-d_6

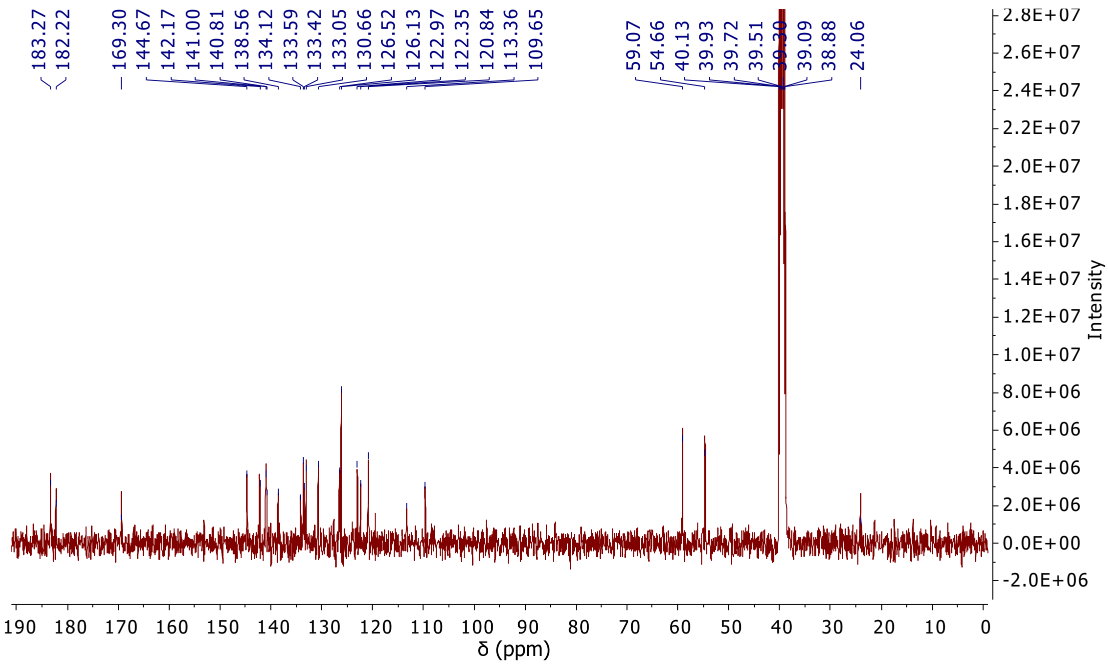

^{13}C NMR (101 MHz, DMSO d_6):δ = 183.27 (s, 1C, C-10); 182.22 (s, 1C, C-13); 169.30 (s, 1C, C-8); 144.67 (s, 1C, C-16); 142.17 (s, 1C, C-18); 141.00 (s, 1C, C-3); 140.81 (s, 1C, C-5); 138.56 (s, 1C, C-11); 134.12 (s, 1C, C-12); 133.59 (s, 1C, C-28); 133.42 (s, 1C, C-27); 133.05 (s, 1C, C-6); 130.66 (s, 1C, C-20); 126.52 (s, 1C, C-21); 126.13 (s, 2C, C-29, C-26); 123.97 (s, 1C, C-4); 122.35 (s, 1C, C-2); 120.84 (s, 1C, C-1); 113.36 (s, 1C, C-17); 109.65 (s, 1C, C-19); 59.07 (s, 1C, C-35); 54.66 (s, 1C, C-36); 39.51 (sept, DMSO d_6); 24.05 (s, 1C, C-24).

Figure S5. ^{13}C-NMR (101 MHz) of acetylated Remazol Blue in DMSO-d_6

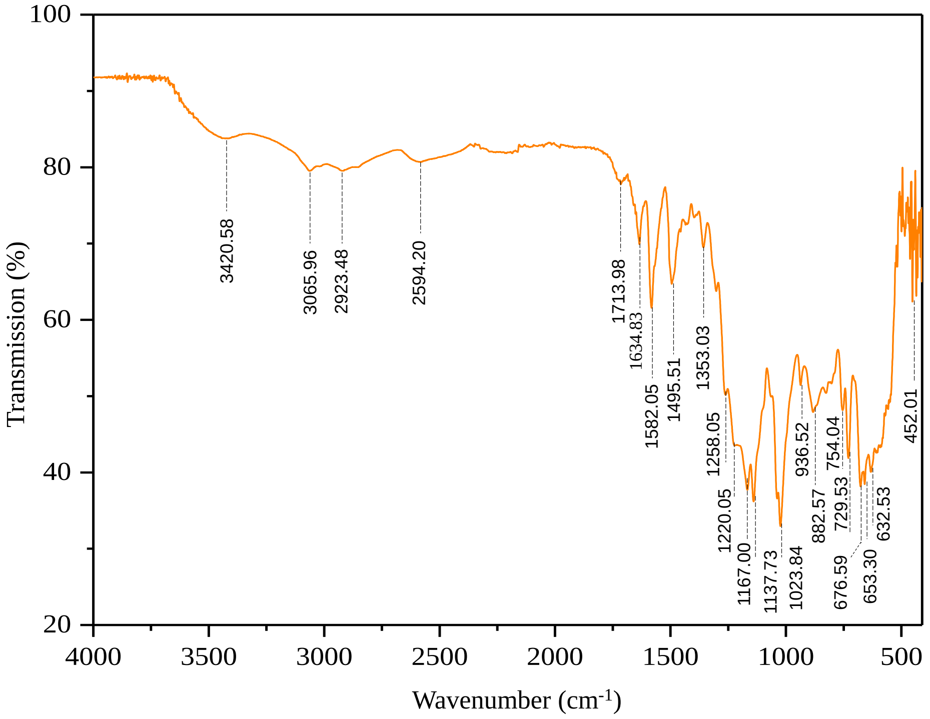

Figure S6. ATR-IR of acetylated Remazol Blue

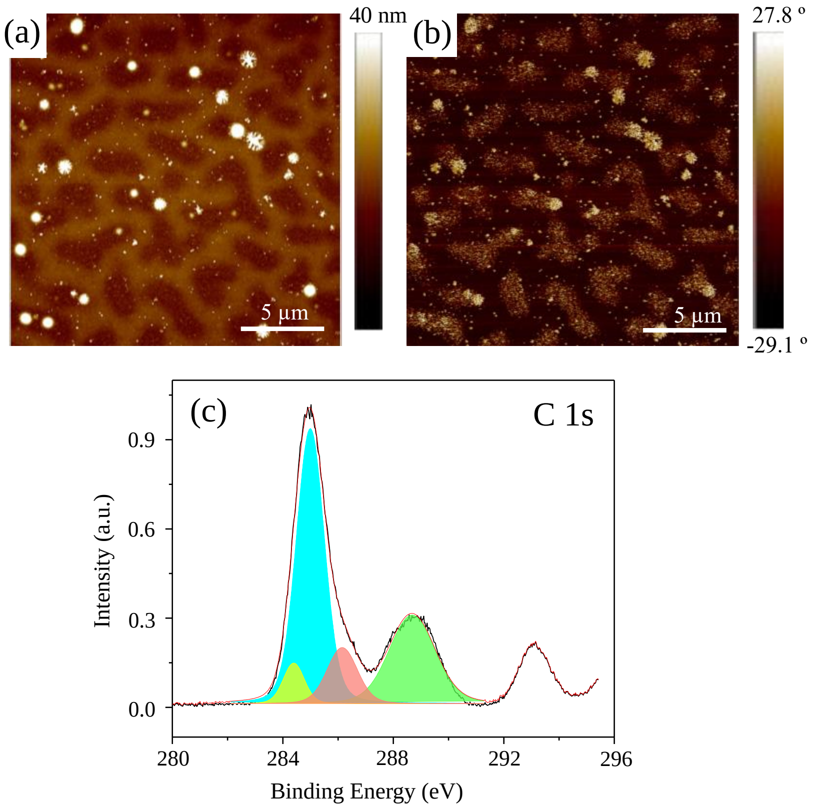

Figure S7. AFM images of (a) the HRP-immobilized surface, along with (b) the corresponding phase image. (c) high-resolution XPS C 1s spectrum of HRP

Table S2. Quantitative immobilization parameters and catalytic performance of the free HRP and immobilized HRP systems:

**Parameter**	**Free HRP**	**Stepwise RB (Directed)**	**PEMA+RB (Directed)**	**Non-Directed (Random)**
Initial Protein Loading (μg)	N/A	300	300	300
Protein Loading on Surface after immobilization ($\mu g \cdot cm^{-2}$)	N/A	0.35	0.21	0.70
Specific Activity ($U \cdot mg^{-1}$)	1.087	0.048	0.044	0.022
Expressed Activity (%)	100	4.4	4.05	2.02
